# MyoD is required for the differentiation of Myo/Nog cell progenitors of myofibroblasts in explants of human lens tissue

**DOI:** 10.3389/fopht.2025.1618276

**Published:** 2025-08-04

**Authors:** Jacquelyn Gerhart, Mara Crispin, Brian Heist, Keith Mathers, Joseph Infanti, David Venuti, Joseph F. Richards, Steven Morency, Cathy Hatcher, Mindy George-Weinstein

**Affiliations:** ^1^ Division of Research, Philadelphia College of Osteopathic Medicine, Philadelphia, PA, United States; ^2^ Main Line Surgery Center, Bala Cynwyd, PA, United States; ^3^ Department of Biomedical Sciences, Philadelphia College of Osteopathic Medicine, Philadelphia, PA, United States

**Keywords:** posterior capsule opacification, lens, myofibroblasts, MyoD, Myo/Nog cells

## Abstract

**Introduction:**

Posterior capsule opacification (PCO) is a complication of cataract surgery that impairs vision. Clouding and distortion of the posterior capsule occur as a result of cell migration, deposition of extracellular matrix proteins and contractions of myofibroblasts. The focus of this study is a subpopulation of cells within the lens, called Myo/Nog cells, that differentiate into myofibroblasts in response to wounding. Myo/Nog cells express the skeletal muscle specific transcription factor MyoD, bone morphogenetic protein inhibitor Noggin and brain-specific angiogenesis inhibitor (BAI1). Depletion of Myo/Nog cells in explants of human anterior lens tissue and the rabbit lens during cataract surgery prevented the emergence of myofibroblasts, and in the rabbit, reduced PCO and anterior capsule opacification to below clinically significant levels. A requirement for MyoD in the differentiation of Myo/Nog cells to myofibroblasts was explored in explant cultures of human anterior lens tissue removed during cataract surgery.

**Methods:**

Human anterior lens tissue was removed by femtosecond laser capsulotomy or curvilinear capsulorhexis during cataract surgery. Tissue was incubated in serum free DMEM/F12 medium for five days with MyoD siRNA, non-targeting siRNA or siRNA delivery buffer. In situ hybridization was carried out with fluorescent probes for human MyoD and Noggin mRNAs. MyoD, Myf5, Myogenin, alpha smooth muscle actin (α-SMA), striated muscle myosin heavy chain and Ki67 proteins were localized by immunofluorescence localization. Cell free areas of the capsule were identified by differential interference and fluorescence microscopy.

**Results:**

Approximately seven percent of the cells in control cultures co-expressed MyoD and Noggin mRNAs. The number of MyoD mRNA-positive (+) cells was reduced by 90% after treatment with MyoD siRNA. The Noggin mRNA+ population was significantly increased with MyoD knockdown. Nearly all cells with BAI1 contained MyoD protein and all had Noggin protein. The MyoD family members Myf5 and Myogenin were also synthesized in Myo/Nog cells. More BAI1+ cells contained Myf5 than Myogenin. The majority of cells with BAI1 synthesized α-SMA and striated muscle myosin. Incubation with MyoD siRNA nearly eliminated Myogenin and striated muscle myosin, and significantly reduced the number of BAI1+ cells with Myf5. Expression of α-SMA was unaffected by MyoD knockdown. The numbers of BAI1+ and BAI1-negative (-) lens epithelial cells (LECs) increased in response to treatment with MyoD siRNA. Noggin and muscle proteins were not detected in LECs in control explants or after MyoD knockdown.  Wounds, defined as areas of the capsule denuded of cells, were surrounded by Myo/Nog cells containing muscle proteins in control cultures. Wrinkles in the capsule were visible within most wounds. BAI1+/α-SMA+ cells continued to form a rim around wounds, but wrinkles were reduced by approximately 75% after MyoD knockdown.

**Discussion:**

These results indicate that MyoD lies upstream of Myogenin, impacts Myf5 expression and is required for striated muscle myosin synthesis in Myo/Nog cells of the lens. Contractions that deform the anterior capsule are dependent on striated muscle myosin but not α-SMA. Overall, this study demonstrates that MyoD drives Myo/Nog cell differentiation to contractile myofibroblasts in primary cultures of human anterior lens tissue. While this study defines an obligatory mechanism for Myo/Nog cell differentiation, the resulting increase in the progenitor population indicates that temporary knockdown of MyoD is not a therapeutic option for preventing of PCO.

## Introduction

Posterior capsule opacification (PCO) is a condition that impairs vision in greater than 20% of adults and nearly all children following cataract surgery (1–3). Clouding of the posterior capsule occurs as a result of cell migration and deposition of exrtracellular matrix (ECM) proteins (3, 4). An outcome of surgery that also significantly impacts vision in PCO is the emergence of myofibroblasts whose contractions deform the lens capsule (4–6).

Myofibroblasts have been reported to arise from lens epithelial cells (LECs) that undergo an epithelial to mesenchymal transition (EMT), migrate onto the posterior capsule and initiate transcription of alpha smooth muscle actin (α-SMA), a marker of transdifferentiation (3–6). We have identified a subpopulation in the lens, called Myo/Nog cells, that are inherently myogenic, migratory and directly differentiate into myofibroblasts (7–10). Myo/Nog cells were named for their expression of the skeletal muscle-specific transcription factor MyoD and bone morphogenetic protein (BMP) inhibitor Noggin (7, 11–13). Another marker of these cells is brain-specific angiogenesis inhibitor 1 (BAI1) recognized by the G8 monoclonal antibody (mAb) (12, 14–16). Myo/Nog cells were first discovered in the blastocyst of the chick embryo (11, 15, 17). They are integrated in low numbers in muscle and non-muscle forming tissues during gastrulation (13, 18, 19). In the eyes, Myo/Nog cells are present in the lens placode, optic vesicle and surrounding mesoderm, and later in the lens, retina, cornea, ciliary body, iris and extraocular muscles, and on the zonules of Zinn (for review see (7). Depletion of Myo/Nog cells in the blastocyst results in severe malformations of the eyes and a lack of skeletal muscle due to the absence of Noggin and hyperactive BMP signaling (13, 19, 20).

Expression of MyoD mRNA in Myo/Nog cells is associated with their stable commitment to the skeletal muscle lineage (14, 18). BAI1-positive (+) cells isolated from the blastocyst, embryonic brain and heart, and fetal organs rapidly translate MyoD mRNA, synthesize striated muscle proteins, assemble sarcomeres and fuse to form multinucleated myofibers when cultured in serum-free medium (14, 18). In the adult eye, cell death, wounding, hypoxia and light damage trigger Myo/Nog cells to proliferate, migrate, engage in phagocytosis and undergo a partial completion of the skeletal muscle program as they differentiate into single nucleated myofibroblasts lacking sarcomeres (8, 10, 21–26).

Multiple skeletal muscle proteins are expressed in Myo/Nog cells of the lens. In cultures of anterior lens tissue removed from patients during cataract surgery, Myo/Nog cells synthesize MyoD protein, striated muscle myosin heavy chain II, skeletal muscle specific troponin T and the 12101 protein associated with T-tubules, and α-SMA (8, 9). Wounds, defined as areas of the capsule denuded of cells, are surrounded by Myo/Nog cells that deform the capsule (8). Targeted depletion of Myo/Nog cells with the BAI1 monoclonal antibody (mAb) (formerly known as the G8 mAb) and complement, or the BAI1 mAb conjugated to 3DNA nanocarriers intercalated with doxorubicin, prevents the emergence of myofibroblasts in human lens explant cultures (8, 9). This drug also specifically kills Myo/Nog cells, nearly eliminates myofibroblasts and folds in the capsule, and reduces PCO and ACO to below clinically significant levels when injected into the rabbit lens during cataract surgery (10). Lens epithelial cells (LECs) do not transdifferentiate into either Myo/Nog cells or myofibroblasts that express striated muscle proteins following Myo/Nog cell depletion *in vitro* or *in vivo* (8–10). However, LECs do express α-SMA and migrate into a scratch wound in the absence of Myo/Nog cells when human anterior lens tissue is treated with transforming growth factor-beta 1 (TGFß1) (8). Expression of α-SMA in LECs in response to TGFß also was reported by others using the human anterior lens culture system and in rat lens explants (6, 27).

Myofibroblasts are problematic in the retina, as well as the lens. Proliferative vitreoretinopathy (PVR) is characterized by the formation of epiretinal membranes (ERMs), most often in response to repair of a retinal detachment (28–30). Myofibroblasts within ERMs produce a tractional force that may lead to re-detachment and blindness (28, 31, 32). ERMs removed from patients with PVR contain cells in which MyoD, Noggin and BAI1 co-localize with muscle proteins (23). Differentiated Myo/Nog cells accumulate in ERMs overlying retinal folds and areas of detachment in a mouse model of PVR (25).

The above-mentioned *in vitro* and *in vivo* studies demonstrate a correlation between MyoD expression and the differentiation of Myo/Nog cells to myofibroblasts in the lens and retina. MyoD was first discovered by its ability to convert 10T1/2 fibroblasts and other cell types to skeletal muscle myoblasts (33–36). Three other members of the MyoD family, Myf5, Myogenin and MRF4/Myf6, were subsequently identified (37–39). Within the embryo, MyoD and Myf5 promote the commitment of progenitor cells to the skeletal muscle lineage. Although these transcription factors can compensate for the loss of one another to support skeletal myogenesis in the embryo, differentiation is delayed in satellite cells of regenerating muscle in MyoD null mice (40–48). Myogenin knockout mice contain myoblasts but lack functional skeletal muscle, illustrating its role in terminal differentiation (49–51). The mechanisms whereby these transcription factors affect skeletal muscle gene expression differ; Myf5 reorganizes the chromatin, while MyoD and Myogenin initiate transcription (52–54). MRF4 can rescue myogenesis in absence of both Myf5 and MyoD to mediate specification and differentiation (55).

In the following experiments, we utilized human anterior lens tissue removed during cataract surgery to determine whether MyoD is required for Myo/Nog cell differentiation to myofibroblasts and the generation of force that deforms the capsule. The results illustrate relationships between MyoD and other members of the skeletal myogenic transcription factor family (MRFs), muscle protein accumulation, contraction and proliferation.

## Materials and methods

### Procurement of human anterior lens tissue

This research followed the tenets of the Declaration of Helsinki. Written informed consent was obtained from patients undergoing cataract surgery. The study was approved by the Philadelphia College of Osteopathic Medicine’s Institutional Review Board (H17-018).

Anterior lens tissue was obtained by precision pulsed femtosecond laser capsulotomy (91% of cases) or continuous curvilinear capsulorhexis (9% of cases). Tissue was collected in DMEM/F12 medium containing 1% penicillin and streptomycin (Gibco/Life Technologies, Grand Island, NY). The extent of cellular coverage of the capsule was approximately 62% with both methods of tissue extraction.

### Explant cultures of human lens tissue

Lens tissue was cultured in 400 µl of DMEM/F12 without antibiotics in Lab Tek II 8 well Chamber slides (Thermo Fisher Scientific, Waltham, MA) for five days at 37° in 5% CO_2_ in air. Whole, circular pieces of tissue were cut in half and cultured in separate wells. Two days after plating, an additional 100 µl of DMEM/F12 was added to each well. Tissue was fixed in 2% formaldehyde for 10 minutes on day 5.

### Treatment with siRNA

Dharmacon™ Accell™ SMART pool human MYOD1 small interfering RNA (siRNA) consisted of the following sequences: siMyoD1 A-010316–13 UUGUAAUAC UUUUGUAAUC; siMyoD1 A-010316–14 CGGACGACUUCUAUGACGA; siMyoD1 A-010316–15 CGCCUGAGCAAAGUAAAUG; and siMyoD1 A-010316–16 GUGUGGUGCUACAGGGAAU. Sequences targeted with these siRNAs lack homology with Myf5, Myogenin and MRF4 mRNAs. The non-targeting control pool siRNA (NT siRNA; D-001910-10-20) served as a control for the effects of MyoD siRNA. siRNAs were resuspended in RNase-free 1x siRNA buffer (Horizon Discovery, Cambridge, UK) and quantified by UV spectrophotometry according to the manufacturer’s instructions. Four ng of siRNA in 4 µl of 1x siRNA buffer were added to each well at the time of plating. Additional controls included no treatment and 1x siRNA buffer only.

### *In situ* hybridization

*In situ* hybridization was carried out with probes for human MyoD (#562721) and Noggin (#416521) using the RNAscope™ 2.5 HD Duplex Reagent Kit (Advanced Cell Diagnostics, Inc., Neward, CA). The MyoD and Noggin chromogenic probes were conjugated with the Fast Red and horseradish peroxidase (HRP-green), respectively. A probe for *Bacillus subtilis* with the Fast Red chromagen (dapB, ACD #PN31004) was used as a negative control.

### Immunofluorescence localization

Fixed tissue was rinsed in phosphate buffered saline (PBS) and permeabilized in 0.1% Triton X-100. Double labeling was carried out as described previously (11). Primary antibodies included: 1) mouse mAbs to BAI1 (14), MyoD (MA5-12902, Invitrogen/Thermo Fisher Scientific), Myogenin (F5D Developmental Studies Hybridoma Bank, Iowa), α-SMA (F3777; Sigma -Aldrich, St. Louis, MO) and striated muscle myosin heavy chain (ab58899; Abcam, Cambridge, MA); 2) rabbit mAbs to Myogenin (Ab124800; Abcam, Cambridge,MA) and the proliferation marker Ki67 (ab16667; Abcam); 3) a goat polyclonal antiserum to Noggin (AF71; R&D BioTechne, Minneapolis, MN); and 4) a rabbit polyclonal antiserum to Myf5 (PA5-115608; Invitrogen ThermoFisher). Primary antibodies were visualized with species and subclass specific AffiniPure Fab fragments conjugated with Rhodamine Red or Alexa 488 (Jackson Immunoresearch, West Grove, PA). The α-SMA mAb was directly conjugated with fluorescein. The muscle myosin heavy chain antibody did not bind smooth muscle of the vasculature (25). An additional antigen retrieval step of 0.5% Triton at 37°C for 10 minutes was added for labeling with the Myogenin mAb. Explants were transferred to glass slides and coverslips applied with Fluoro-Gel II mounting medium containing 4, 6-diamino-2-phenylindole (DAPI) (Electron Microscopy Sciences, Hatfield, PA).

### Fluorescence analyses and photography

Quantitation of fluorescently labeled cells was carried out with the Nikon Eclipse E800 epifluorescence microscope (Nikon Instruments Inc., Melville, NY) equipped with 10X, 60X and 100X lenses, the Infinity 3S camera (Teledyne DALSA, Waterloo, Ontario, Canada) and the Image Pro Plus image analysis software program (Media Cybernetics, Rockville, MD). The numbers of unlabeled and single and double labeled cells were determined in a minimum of 20 fields containing a minimum of 200 cells by a previously validated sampling method (8). Imaging was also carried out with the Olympus Fluoview 1000 and Evident Scientific Fluoview 4000 confocal microscopes equipped with a 60x oil immersion lens and Fluoview software programs (Olympus Corp. Tokyo, Japan; Evident Scientific, Waltham, MA). Images were adjusted for brightness and contrast, and figures assembled in Adobe Photoshop version 23 (Adobe Inc., San Jose, CA).

### Quantitation of wounds and wrinkles in the capsule

Wounds are defined as circular to oval areas denuded of LECs. Deformations of the capsule within wounds appear as wrinkles. The number of wounds with and without wrinkles in each explant was quantified using the Nikon Inverted Eclipse Ti microscope and differential interference contrast (DIC) optics. Deformations of the capsule were also analyzed outside of the wounds in areas of the explants covered by LECs. For this analysis, the tissue was placed on the slide with the capsule facing the coverslip. Wrinkles were photographed with a Nikon Ci-L light microscope or the Olympus Fluoview 1000 confocal microscope.

### Statistical analyses

Percentages, means and standard deviations (SDs) were calculated for each explant and culture condition. Statistical analyses were conducted using SigmaPlot v14.0. The following statistical tests were applied: the t-test and Mann-Whitney test for comparing two conditions, Analysis of Variance (ANOVA) for comparing means among three or more groups, and the Kruskal-Wallis test for analyzing differences among multiple groups when data do not meet parametric assumptions. For pairwise comparisons, Tukey’s test was used following ANOVA, while the Dunn’s test was employed following the Kruskal-Wallis test. The Mann-Whitney and Kruskal-Wallis tests were used when the data did not meet the assumptions of normality and/or equal variance. A p-value of ≤ 0.05 was considered significant.

## Results

### Treatment with MyoD siRNA reduces MyoD but not Noggin mRNA in lens explants

**Figure 1:**
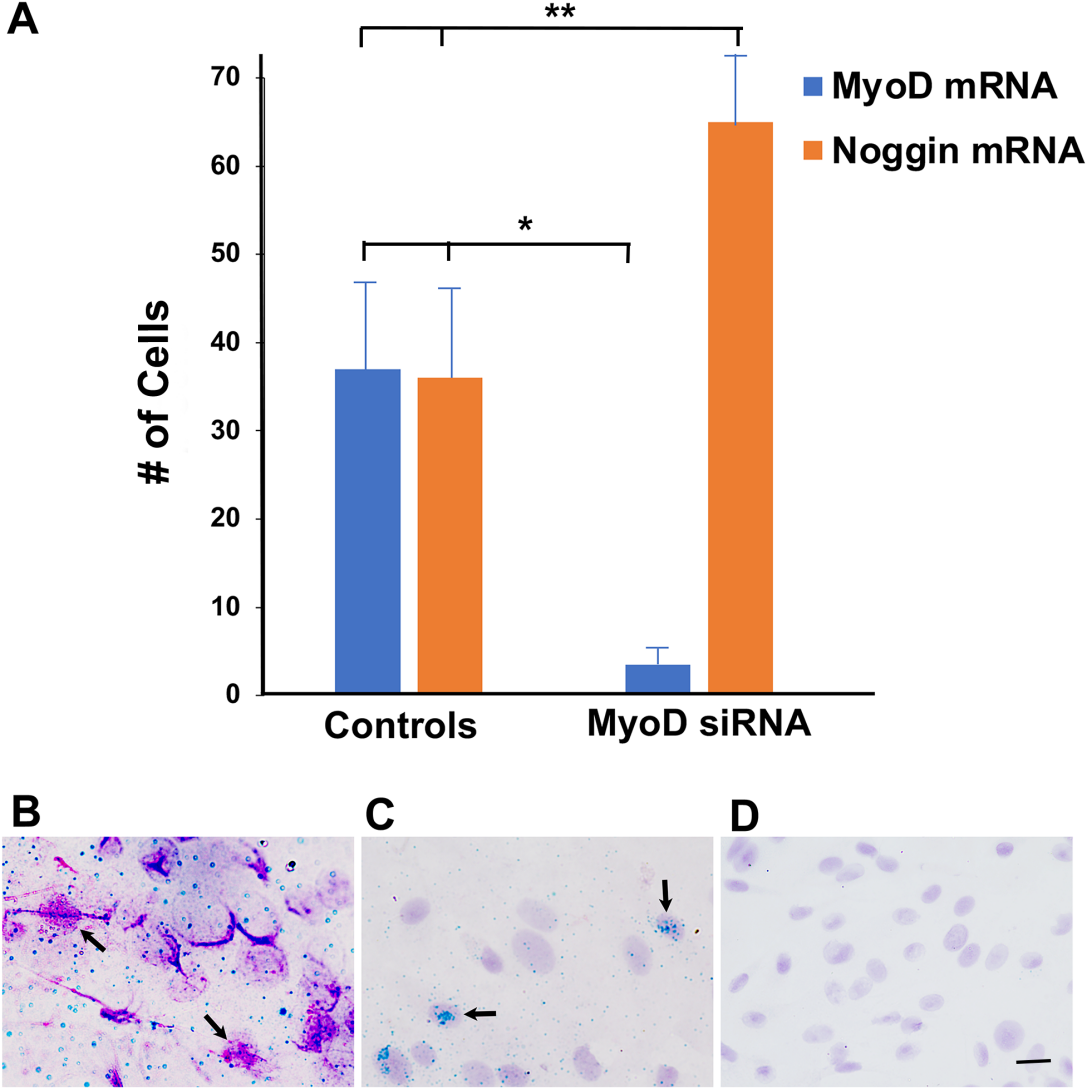
Effect of MyoD siRNA on the expression of MyoD and Noggin mRNAs in lens explant cultures. Lens explants were untreated or incubated with buffer, NT siRNA (Controls) or MyoD siRNA for 5 days. Tissue was probed for MyoD mRNA (Fast Red) and Noggin mRNA (HRP-green). **(A)** Single and double labeled cells were counted in a minimum of 20 fields/explant. Control data consisted of results pooled from three untreated, two buffer and three NT siRNA cultures for MyoD, and two untreated, one buffer and three NT siRNA for Noggin. Six explants were incubated with MyoD siRNA. Single and double labeled cells were counted in a minimum of 20 fields in each explant. The results are the mean and standard deviation. Treatment with MyoD siRNA significantly reduced the numbers of cells with MyoD mRNA (*p < 0.0001) and increased cells with Noggin mRNA (**p = 0.001) compared to controls. **(B)** An explant incubated with NT siRNA for five days contained cells double labeled for MyoD and Noggin mRNAs (arrows). **(C)** An explant incubated with MyoD siRNA contained cells with Noggin but not MyoD RNA (arrows). **(D)** A Fast Red probe for *Bacillis subtilus* did not label cells in an explant treated with MyoD siRNA. Bar = 15 µM in **(B, C)**, and 10 µM in **(D)**.

The effect of MyoD siRNA on MyoD mRNA expression was determined by qualitative *in situ hybridization* on the fifth day in culture. In control cultures, 7 ± 1% of cells (n = 8) were labeled with the Fast Red probe for MyoD mRNA. The number of cells with MyoD mRNA was reduced by 90% after treatment with MyoD siRNA for five days (**Figure 1A**).

A probe for Noggin mRNA was used as a control for the specificity of MyoD siRNA. In control cultures, Noggin and MyoD mRNAs were co-localized in 96 + 7% of cells with MyoD mRNA (**Figure 1B**). A subpopulation of cells continued to expressed Noggin mRNA in the absence of MyoD (**Figure 1C**). The number of Noggin mRNA-positive (+) cells was increased in response to MyoD knockdown (**Figure 1A**). There was no labeling with the negative control probe for *Bacillis subtilus* (**Figure 1D**).

### BAI1+ cells co-express Noggin but not MyoD protein after treatment with MyoD siRNA

**Figure 2:**
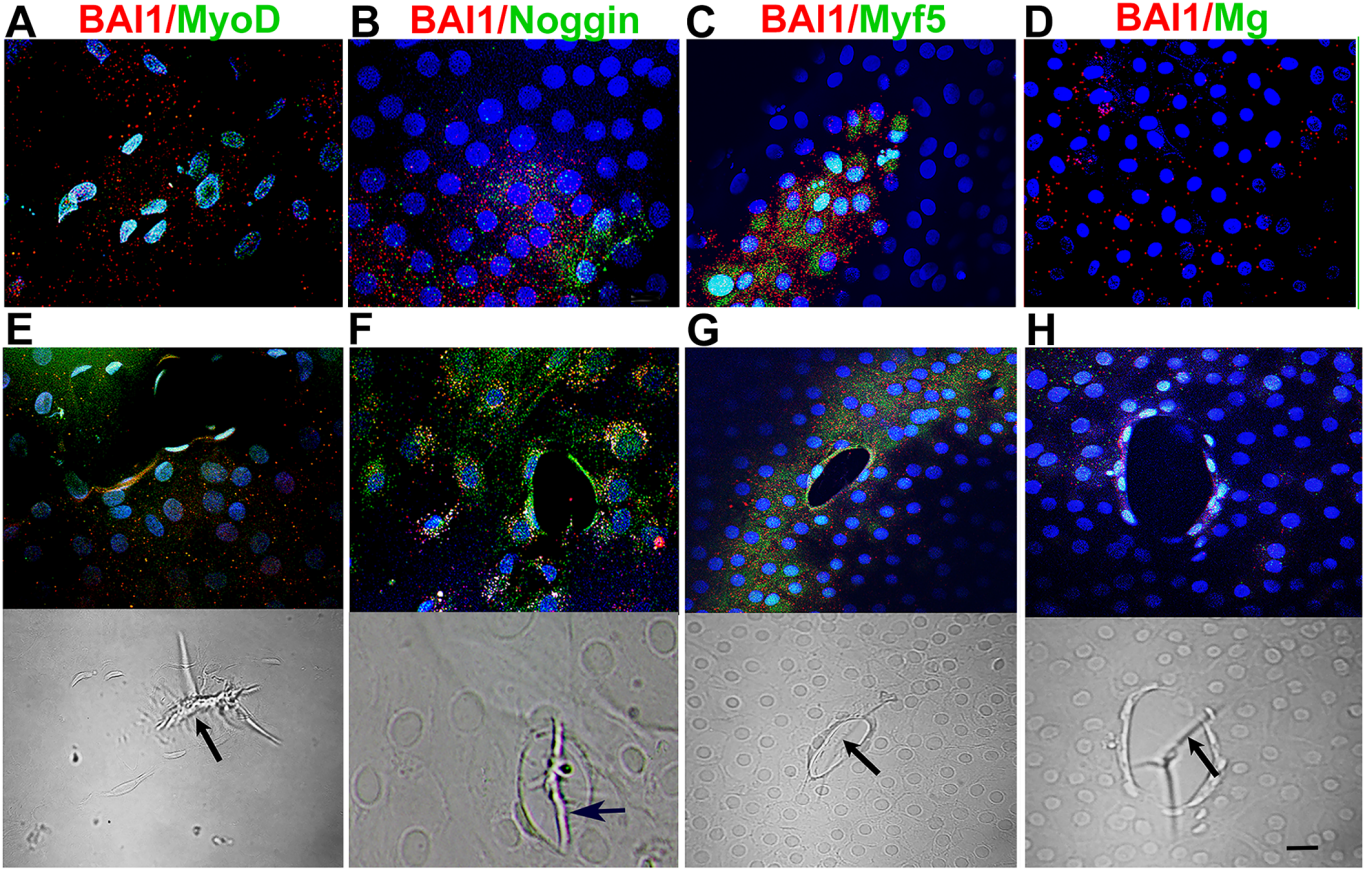
Distribution of Myo/Nog cells with MRF and Noggin proteins in 5-day control cultures. Explants in control cultures were double labeled with antibodies to BAI1 (red) and MyoD, Noggin, Myf5 and Myogenin (green). Nuclei were labeled with DAPI (blue). Labeled cells are shown between wounds **(A–D)** and surrounding wounds in the epithelium **(E–H)**. Fluorescence and DIC images are included for each wound. BAI1+ cells between and surrounding wounds contained Myf5 and Noggin proteins **(A, B, D)**. Fewer BAI1+ Myo/Nog cells had Myogenin protein between wounds than surrounding them **(C, D)**. Wrinkles in the capsule were present within the wounds [arrows in DICs **(E-H)**]. Bar = 10 µM.

**Figure 3:**
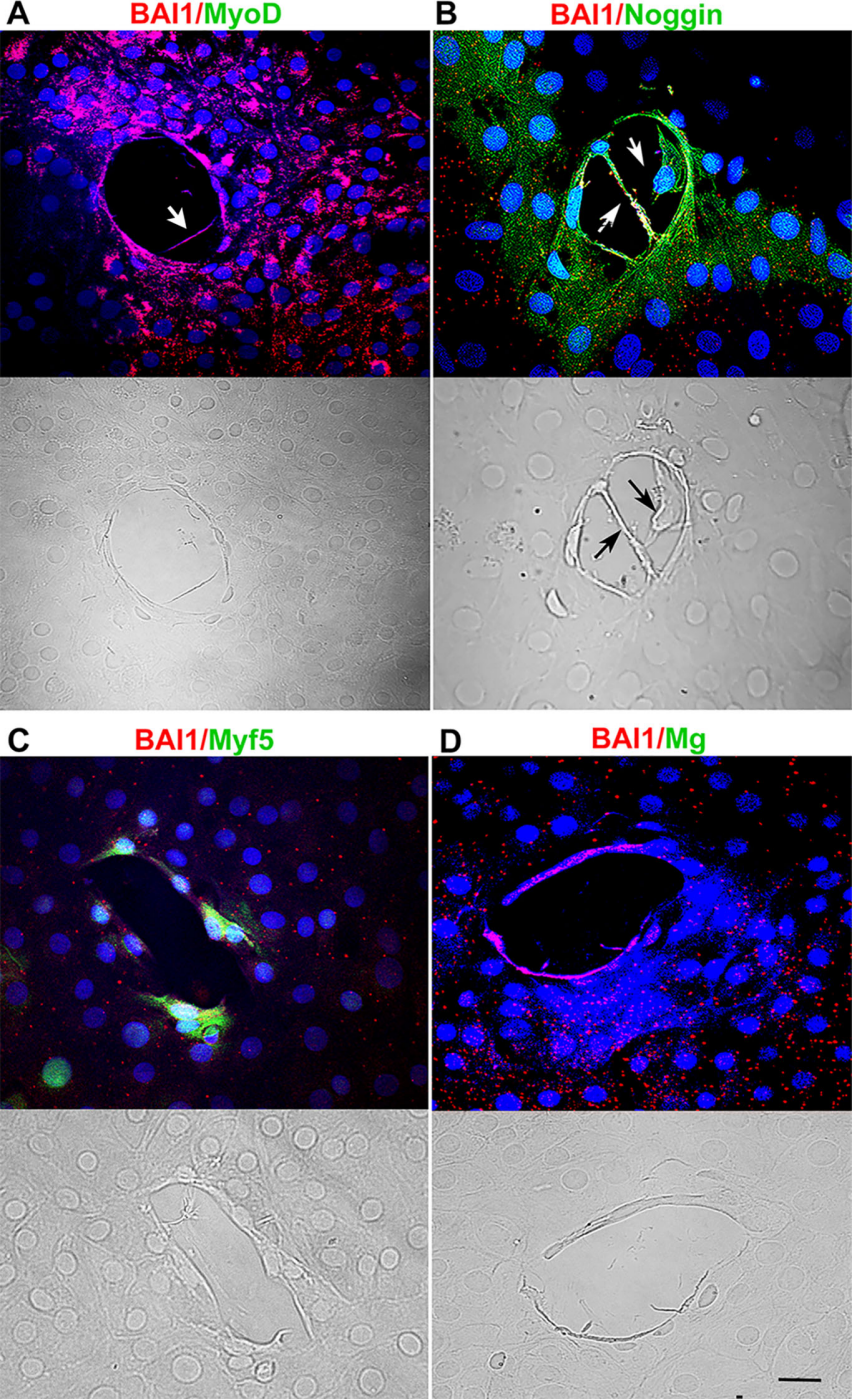
Effect of MyoD knockdown on MRF and Noggin proteins, and deformations of the lens capsule. Explants were incubated with MyoD siRNA for five days and double labeled with antibodies to BAI1 (red) and MyoD, Myf5, Myogenin or Noggin (green). Nuclei were stained with DAPI (blue). Fluorescence and DIC images are shown for each wound. MyoD and Myogenin were not detected in BAI1+ cells surrounding the wounds **(A, C)**. Myf5 was present in some BAI1+ cells **(B)**. All BAI1+ cells contained Noggin **(D)**. Wrinkles in the capsule were absent within the wounds. Cells and their processes were present within the wound (arrows in A and B). Bar = 10 µM.

The third specific marker of Myo/Nog cells is BAI1 (12, 14–16). Double label immunofluorescence localization was performed to examine co-expression of BAI1 with MyoD and Noggin in the presence and absence of MyoD siRNA. As expected, practically all BAI1+ cells contained MyoD protein and all had Noggin protein in control cultures (**Table 1**), thus confirming their identity as Myo/Nog cells. Cells with both proteins were similarly distributed throughout the explant as those with MyoD and Noggin mRNAs (**Figures 1**, **2**). Explants treated with MyoD siRNA contained very few BAI1+ cells with MyoD protein (**Table 1**; **Figure 3**). Expression of Noggin protein in BAI1+ cells did not change with MyoD knockdown (**Table 1**; **Figures 2**, **3**).

**Table 1:** Co-localization of BAI1 with Noggin and MRF proteins in 5-day lens cultures.

Percent	No Tx	Buffer	NT siRNA	MyoD siRNA
% BAI1+ with MyoD	100 (5)	99 ± 2 (5)	96 ± 3 (5)	1 ± 1 (5)
% MyoD+ with BAI1	100	100	100	100
% BAI1+ w/Noggin	100 (4)	100 (5)	100 (4)	100 (4)
% Noggin+ w/BAI1	100	100	100	100
% BAI1+ w/Myf5	80 ± 23 (5)	65 ± 20 (5)	81 ± 14 (5)	19 ± 16 (5)
% Myf5+ w/BAI1	99 ± 2	100	100	100
% BAI1+ w/Myogenin	19 ± 11 (5)	45 ± 11 (5)	35 ± 13 (5)	2 ± 1 (5)
% Myogenin+ w/BAI1	100	100	100	100

Explants were cultured for five days without treatment (No Tx) or in the presence of buffer, NT or MyoD siRNAs. Double labeling was performed with the BAI1 mAb and antibodies to MyoD, Noggin, Myf5 or Myogenin. % BAI1+ cells with MyoD, Myf5, Myogenin and Noggin = (number of BAI1+ cells co-labeled with the other antibody ÷ total BAI1+ cells) X 100. This formula was also used to calculate the percent of MyoD, Myf5, Myogenin and Noggin+ cells that were BAI1+. The number of explants scored is indicated in parenthesis. The results are the mean ± standard deviation. In control cultures, nearly all BAI1+ cells contained MyoD, all contained Noggin and most were labeled for Myf5. More BAI1+ cells contained Myf5 than Myogenin (p value range for all conditions = 0.006-0.001). With rare exception for Myf5 in untreated cultures, the MRFs were present only in BAI1+ cells under all conditions. Treatment with MyoD siRNA nearly eliminated MyoD (p value range compared to controls = 0.0006-0.0001) and Myogenin proteins (p value range = 0.086-0.0001), and significantly reduced Myf5 (p value range = 0.006-0.001).

### MyoD knockdown reduces Myf5 and Myogenin proteins in BAI1+ cells

As was the case for MyoD and Noggin, BAI1+ cells with Myf5 and Myogenin proteins were present throughout the explant but were concentrated in small clusters, larger aggregates and around wounds in the epithelium in control cultures (**Figures 2C, D, G, H**). Most BAI1+ cells contained Myf5 (**Table 1**). Less than half of the Myo/Nog cell population contained detectable levels of Myogenin (**Table 1**). With a rare exception for Myf5, MRFs were detected only in cells with BAI1 under all conditions (**Table 1**). BAI1+/Myogenin+ cells were more prevalent around wounds than in non-wounded areas of the tissue (**Figures 2D, H**).

Treatment with MyoD siRNA nearly eliminated Myogenin and significantly reduced Myf5 (**Table 1**; **Figures 3C, D**). Fewer Myf5+ cells were present around wounds after MyoD knockdown than in control cultures (**Figures 3C**, **2G**). BAI1+ cells and their processes were occasionally observed within the wound (**Figures 3A, B**).

### MyoD knockdown reduces muscle myosin heavy chain, but not α-SMA, in BAI1+ cells

**Figure 4:**
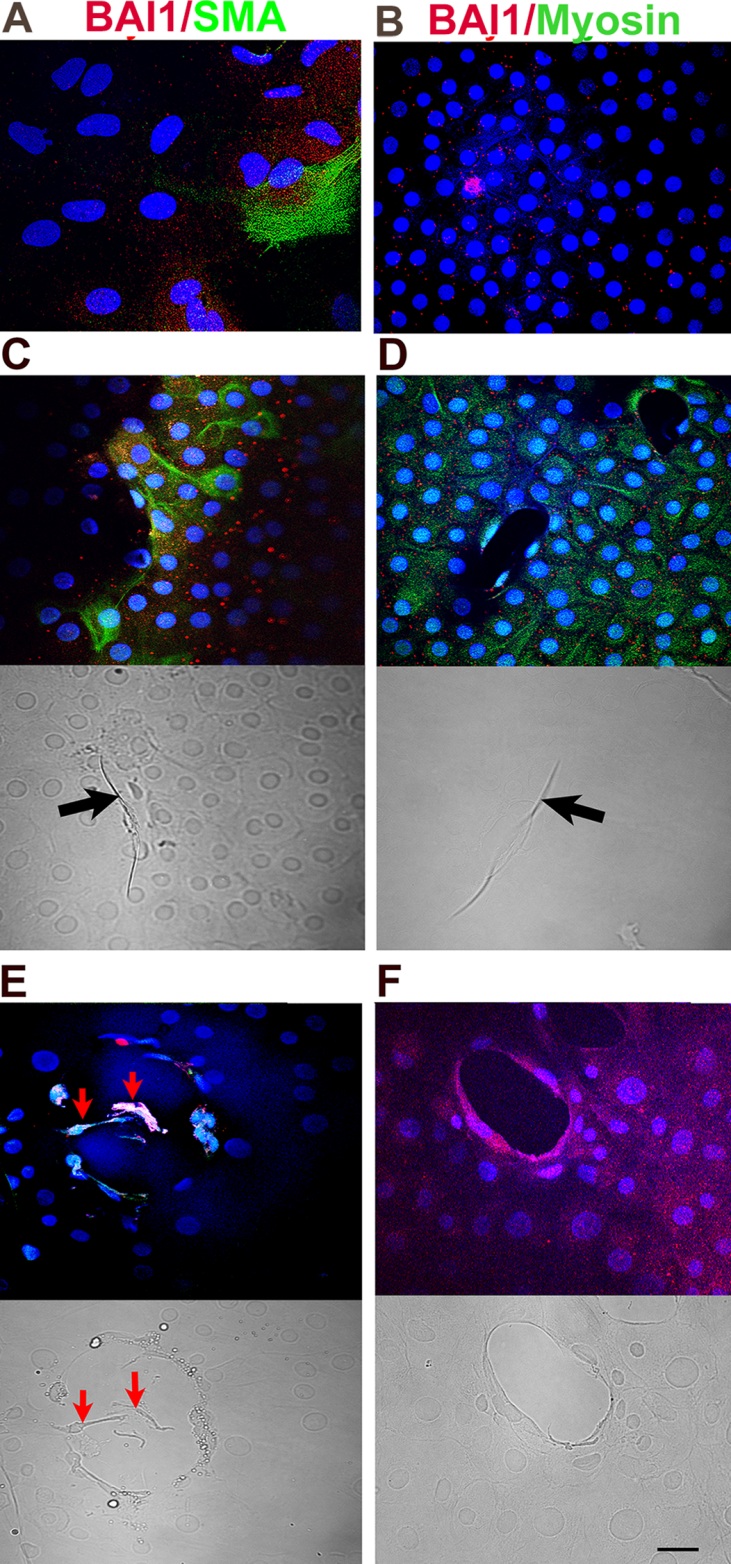
Effect of MyoD knockdown on α-SMA and muscle myosin proteins, and deformations of the lens capsule. Explants in control **(A–D)** and MyoD siRNA treated cultures **(E, F)** were double labeled with antibodies to BAI1 (red) and α-SMA or striated muscle myosin (green) on day 5. Overlap of red and green appears yellow in merged images. Nuclei were stained with DAPI (blue). Labeled cells are shown between wounds **(A, B)** and surrounding wounds in the epithelium **(C–F)**. Fluorescence and DIC images are included for each wound. BAI1+ cells surrounding the wounds expressed α-SMA and striated muscle myosin proteins in control cultures **(C, D)**. α-SMA, but not striated muscle myosin, was present in BAI1+ cells after MyoD knockdown **(E, F)**. A few α-SMA+ cells had migrated into the wound of a MyoD siRNA treated explant (red arrows in E). Wrinkles in the capsule were present within the wounds in control cultures [arrows in **(C, D)**] but not in those treated with MyoD siRNA **(E, F)**. Bar = 10 µM.

α-SMA and muscle myosin heavy chain were expressed exclusively in BAI1+ Myo/Nog cells in lens explants (**Table 2**) and were enriched around wounds in the epithelium (**Figures 4A–D**). The percentages of cells with these muscle proteins did not differ significantly in buffer and NT siRNA treated cultures; however, significantly more BAI1+ cells had α-SMA than muscle myosin in untreated explants than the other two controls. Knockdown of MyoD did not affect the expression of α-SMA, but nearly eliminated labeling for muscle myosin heavy chain (**Table 2**; **Figures 4E, F**). A few BAI1+ cells with α-SMA had migrated onto the capsule within the wounds of MyoD siRNA treated explants (**Figure 4E**).

**Table 2:** Co-localization of α-SMA and muscle myosin heavy chain in BAI1+ cells in 5-day lens cultures.

Percent	No Tx	Buffer	NT siRNA	MyoD siRNA
% BAI1+ w/α-SMA	87 ± 4 (6)	63 ± 12 (5)	80 ± 11 (5)	65 ± 32 (6)
% α-SMA w/BAI1	100	100	100	100
% BAI1+ w/Myosin	47 ± 18 (5)	63 ± 18 (6)	55 ± 25 (5)	1 ± 1 (5)
% Myosin+ w/BAI1	100	100	100	100

Explants were cultured for five days without treatment (No Tx) or in the presence of buffer, NT or MyoD siRNAs. Double labeling was performed with the BAI1 mAb and antibodies to α-SMA or muscle myosin heavy chain. % BAI1+ cells with α-SMA or striated muscle myosin = (number of BAI1+ cells co-labeled with the other antibody ÷ total BAI1+ cells) X 100. This formula was also used to calculate the percent of α-SMA+ and muscle myosin+ cells that were BAI1+. The number of explants scored is indicated in parenthesis. The results are the mean ± standard deviation. The majority of BAI1+ cells contained α-SMA and muscle myosin heavy chain in control cultures. The percentages of cells with α-SMA and muscle myosin were similar in buffer and NT siRNA treated cultures. In untreated cultures, more cells had α-SMA than striated muscle myosin (p = 0.0007). α-SMA was not significantly reduced with MyoD siRNA treatment. Few cells with muscle myosin remained after treatment with MyoD siRNA (p value range compared to controls = 0.004-0.0001). These muscle proteins were only detected in BAI1+ cells.

### MyoD knockdown increases the number of BAI1+ cells in lens explants

**Figure 5:**
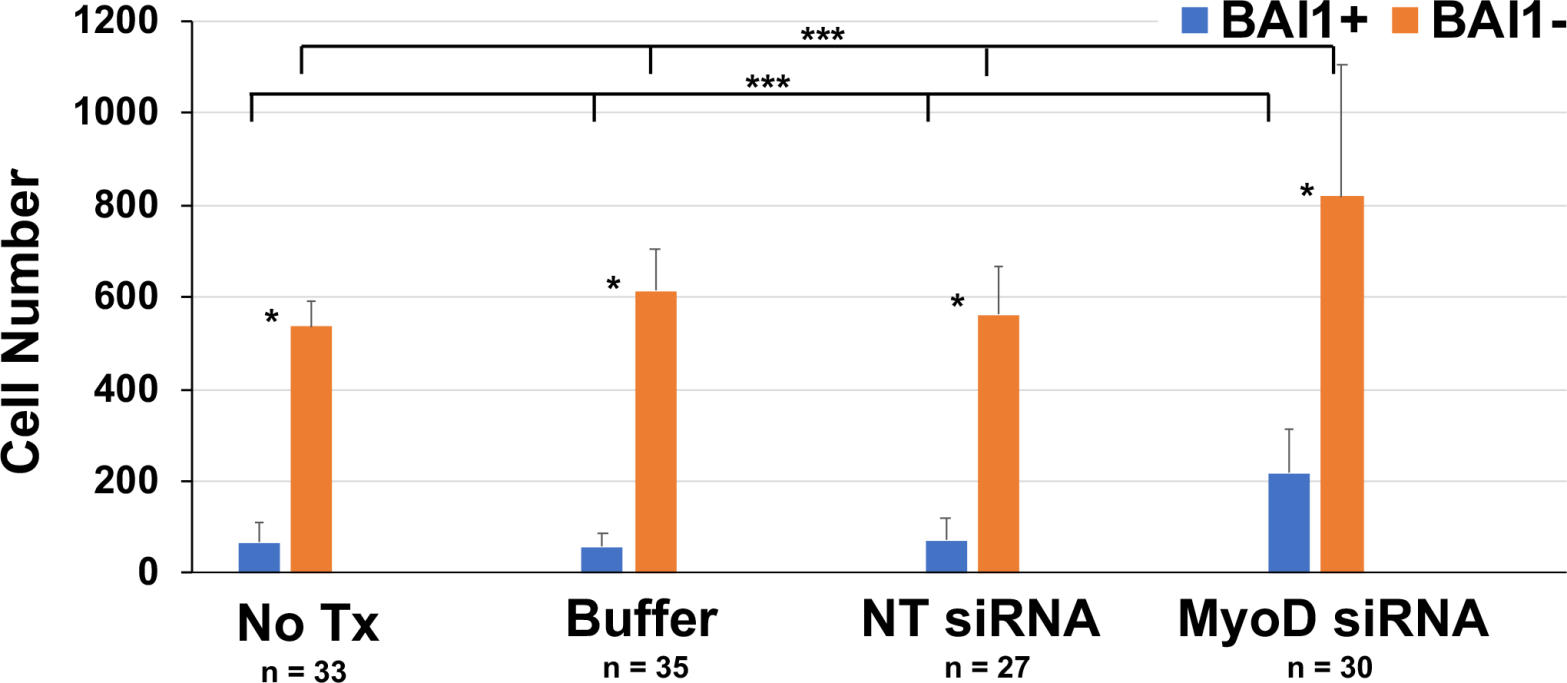
Effect of MyoD knockdown on the numbers of Myo/Nog cells and LECs in lens cultures. Explants were cultured for five days without treatment (No Tx) or in the presence of buffer, NT or MyoD siRNAs. Explants were labeled with the BAI1 mAb on the fifth day in culture. The numbers of BAI1+ and BAI1- cells were counted in a minimum of 20 fields in each explant. n = the number of explants scored. The results are the mean and standard deviation. Significantly more BAI1- than BAI1+ cells were present in explants cultured under all conditions (*p = 0.001). Treatment with MyoD siRNA significantly increased the numbers of BAI1+ (**p < 0.0001) and BAI1- cells (***p = 0.001) compared to control cultures.

Significantly fewer BAI1+ cells were present in lens explants than BAI1-negative (-) LECs under all conditions (**Figure 5**). The relative difference in the populations was reduced after treatment with MyoD siRNA from 8–12 to 3.7 times more LECs than Myo/Nog cells. This reflected a significant increase in the number of BAI1+ cells in the presence of MyoD siRNA and paralleled the increase in cells with Noggin mRNA in response to MyoD knockdown (**Figures 5** and **1**, respectively). The LEC population also increased with MyoD siRNA treatment but to a lesser extent than BAI1+ cells (approximately a 1.5-fold vs. 3.6-fold increase, respectively).

### Subpopulations of cells with and without BAI1 are dividing in 5-day lens explants

**Figure 6:**
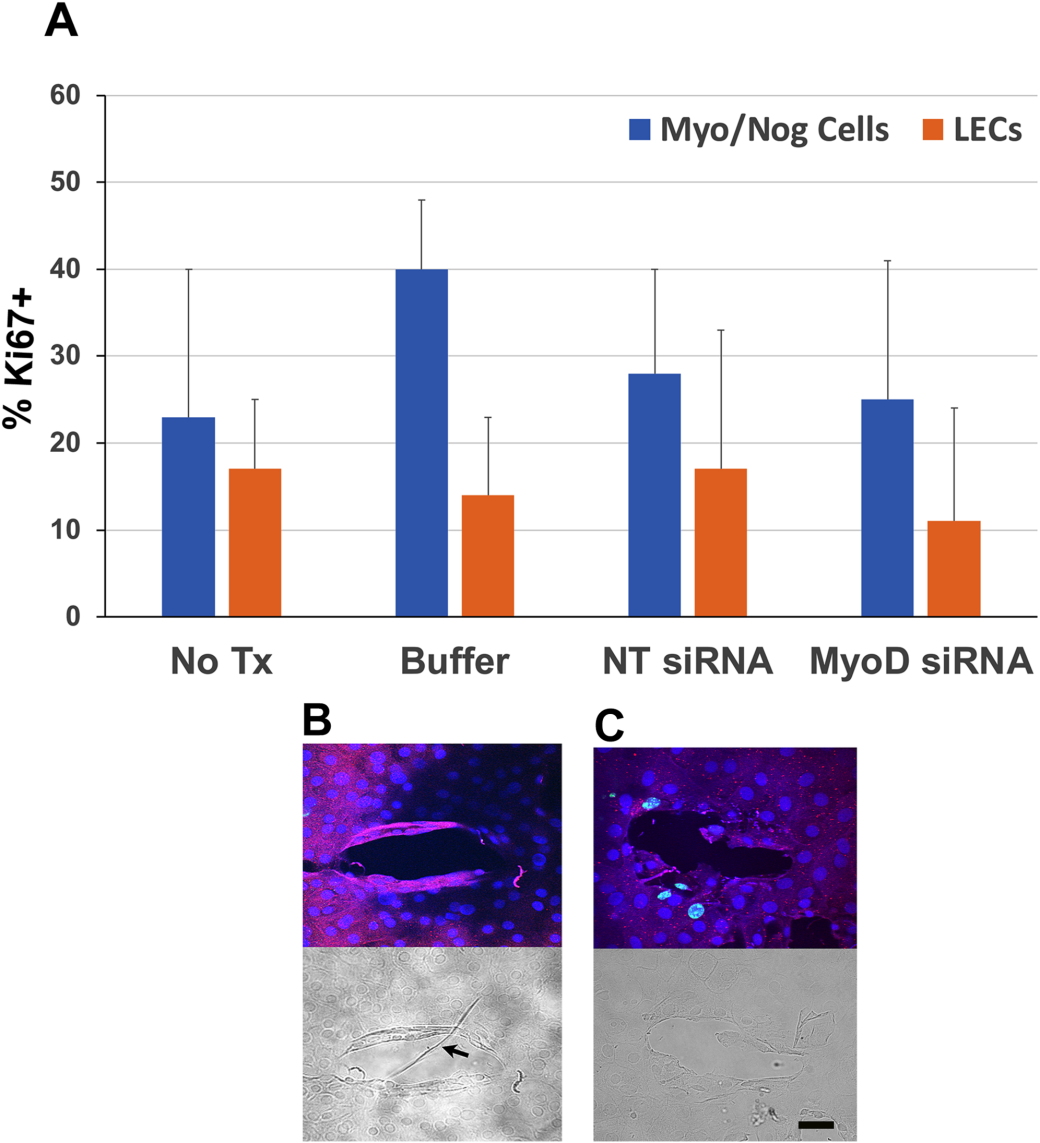
Localization of Ki67 in Myo/Nog cells and LECs in 5-day lens cultures. Explants were cultured for five days without treatment (No Tx) or in the presence of buffer, NT or MyoD siRNAs. Double labeling was performed with antibodies to BAI1 and Ki67 in control and MyoD siRNA treated lens cultures. Nuclei were stained with DAPI. **(A)** % BAI1+ cells with Ki67 = (number of BAI1+ cells co-labeled for Ki67 ÷ total BAI1+ cells) X 100. The % BAI1- cells with Ki67 was also calculated. Five explants were scored for each condition. The results are the mean and standard deviation. Subpopulations of BAI1+ and BAI1- cells were proliferating on the fifth day in culture. No significant differences were found between the percentages of BAI1+ and BAI1- cells with Ki67, or between controls and MyoD siRNA treated cultures. **(B)** BAI1+ cells were Ki67- around wounds in control explants. The DIC image shows a wrinkle in the capsule (arrow). **(C)** Treatment with MyoD siRNA resulted in an increase in BAI1+/Ki67+ cells surrounding the wound without a wrinkle. Bar = 10 µM.

Labeling for Ki67 was used as a marker for all phases of the cell cycle except G0. On average, ≤40% of the BAI1+ and BAI1- cells in lens explants were Ki67+ on day 5 (**Figure 6A**). The percentage of Ki67+ cells did not change significantly when explants were treated with MyoD siRNA. However, the distribution of cycling BAI1+ cells was affected by MyoD knockdown. In control cultures, BAI1+/Ki67+ cells were scarce around the wounds (**Figure 6B**). Treatment with MyoD siRNA resulted in an increase in cycling BAI1+ cells surrounding wounds (**Figure 6C**).

### MyoD knockdown reduces deformation of the lens capsule within wounds

**Figure 7:**
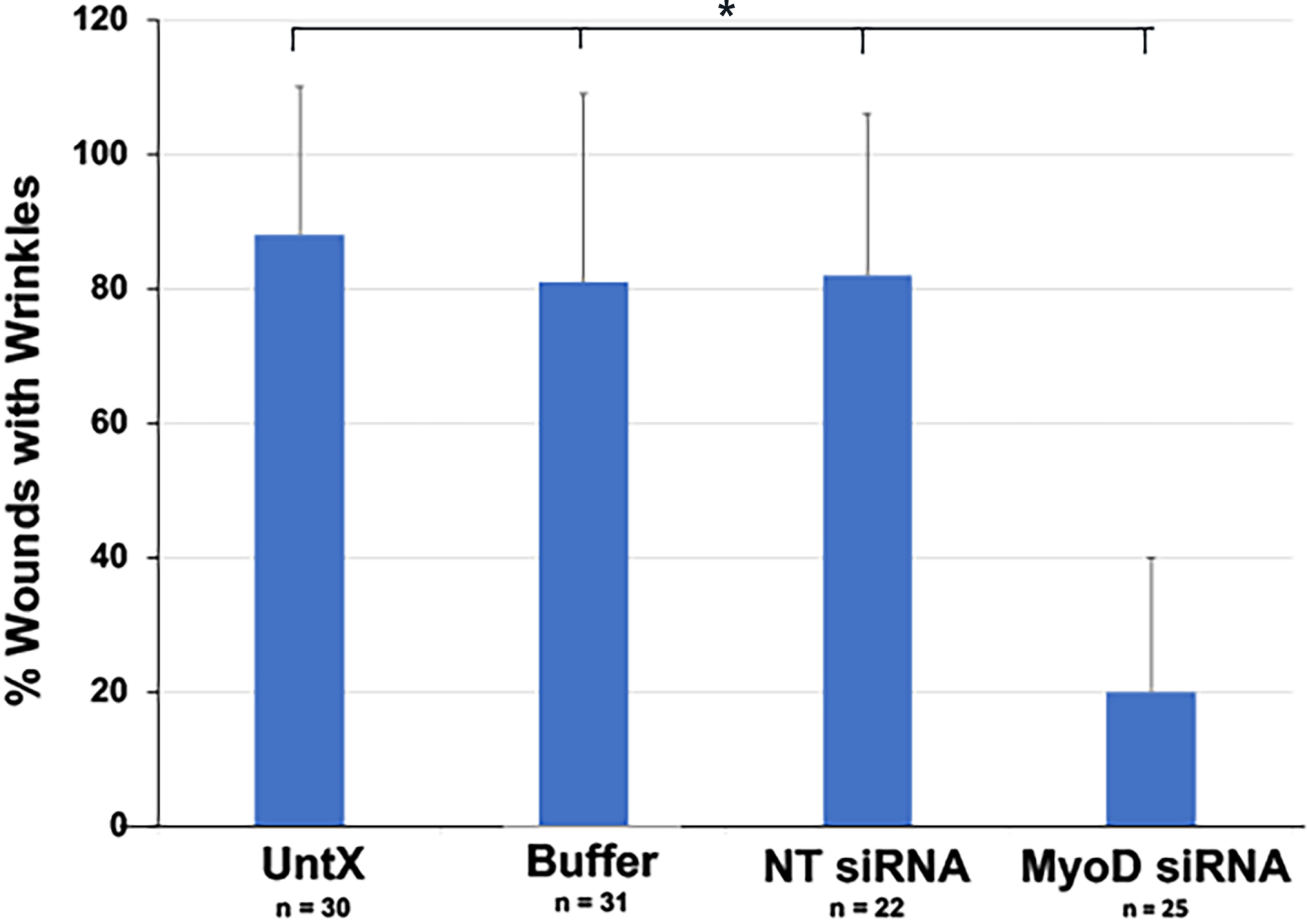
Quantitation of the effect of MyoD knockdown on deformation of the lens capsule within wounds. Explants were cultured for five days without treatment (No Tx) or in the presence of buffer, NT or MyoD siRNAs. The percentage of wounds with wrinkles in the capsule = (number of wounds with wrinkles ÷ total number of wounds) X 100. n = the number of explants scored. The results are the mean and standard deviation. The percentage of wounds with wrinkles in the capsule was significantly less in explants treated with MyoD siRNA than in control cultures (*p < 0.0001).

Wounds were present in 91% of lens explants. The average number of wounds was similar between the four culture conditions (3.3 ± 2.1, n = 117). Most wounds contained a wrinkle in the capsule in control cultures (**Figures 2E–H**, **4C, D**, **7**). Knockdown of MyoD significantly reduced the number of wounds with wrinkles by approximately 75% (**Figure 7**). Areas of the capsule covered by a monolayer of LECs were also screened for deformations of the lens capsule. Wrinkles were not observed outside of the wounds in control or MyoD siRNA treated cultures (not shown).

## Discussion

The goal of this study was to elucidate the role of MyoD in regulating the differentiation of Myo/Nog cells to myofibroblasts in anterior human lens tissue. Cells with detectable levels of MyoD mRNA and protein were nearly eliminated in explants treated with MyoD siRNA. Myogenin protein was also dramatically reduced with MyoD knockdown. This suggests that Myogenin lies downstream of MyoD in Myo/Nog cells of the human lens, a conclusion consistent with analyses of differentiating skeletal myoblasts (56–58). Myf5 protein was significantly reduced with MyoD knockdown but to a lesser extent than Myogenin. The remaining Myf5+ cells were mostly found around wounds where some BAI1+ cells lacking MyoD, Myogenin and muscle myosin heavy chain were proliferating on the fifth day in culture. While Myf5 expression and/or protein stability may be maintained in dividing Myo/Nog cells, this MRF is not upregulated to compensate for the absence of MyoD to support progression towards differentiation as occurs in MyoD null mice (41).

Myofibroblasts derived from other organs synthesize skeletal muscle proteins and their expression is dependent on MRFs (59–69). A more widely used marker for myofibroblasts is α-SMA (4, 6, 28, 29, 60, 70–72). In cultures of rat lung myofibroblasts, expression of α-SMA was dependent on striated muscle myosin-mediated contraction (67). This was not the case in our experiments with human lens explants in which α-SMA protein remained in the near absence of striated muscle myosin and a significant reduction in contractions that deformed the capsule. Dawes et al. demonstrated that knockdown of α-SMA in human lens cell cultures did not affect contraction of a collagen gel (73). These studies indicate that α-SMA alone is not sufficient for contraction of myofibroblasts in lens cultures and striated muscle myosin is required for force generation that deforms the capsule.

Another result of MyoD knockdown in lens tissue explants was an increase in the number of Myo/Nog cells. A similar effect was observed in cultures of human fetal lung myofibroblasts (69). The expansion of the BAI1+ population in the absence of MyoD may be secondary to the reduction in Myogenin; however, both MRFs have been shown to regulate transcription of genes involved in cell cycle withdrawal (74–80). The LEC population also increased in explants treated with MyoD siRNA, although to a lesser extent than Myo/Nog cells. This was an indirect effect because LECs do not express MyoD. An increase in Noggin may indirectly modulate the effects of transforming growth factor beta on LECs in these cultures (8) and further dampen BMP signaling that promotes withdrawal from the cell cycle and differentiation (81). Regulation of proliferation in both populations is likely to involve additional growth factors that may be produced in these serum free cultures.

A subpopulation of Myo/Nog cells was dividing on day 5 in both control and MyoD siRNA treated cultures. While the percentage of cycling Myo/Nog cells was similar among the treatment groups, more Ki67+/BAI1+ cells were observed adjacent to wounds after MyoD knockdown. These wounds were not surrounded by the thickened rim of striated muscle myosin+ cells seen in control cultures. Differentiated Myo/Nog cells immediately surrounding the wound may create a barrier to migration onto the capsule and impose contact inhibition of growth.

Similar to myofibroblasts from other organs, Myo/Nog cells in the lens and retina do not appear to fuse or assemble sarcomeres in response to injury (10, 25). However, they do form multinucleated skeletal muscle myofibers in culture (14, 18). The difference in Myo/Nog cell behavior *in vitro* and *in vivo* may reflect, in part, the mechanism whereby BAI1 mediates myoblast fusion. Engagement of BAI1 with its ligand phosphatidylserine on the surface of apoptotic cells in cultures of skeletal myoblasts stimulates fusion (82). BAI1 null mice have smaller myofibers and impaired muscle regeneration (82). Although BAI1 is expressed in myofibroblasts of the lens and retina (7, 16), there may be too few apoptotic cells within aggregates of differentiating Myo/Nog cells to initiate the fusion process. This also may be the case in other organs in which myofibroblasts activate pro-survival mechanisms (83).

Historically, LECs are considered to be the source of myofibroblasts in the lens (3–6). This conclusion is based on the ability of LECs to undergo an EMT, migrate, synthesize ECM proteins and express α-SMA (3, 4, 6). LECs were thought to be the only population within the lens until the discovery of resident Myo/Nog cells (8, 10, 13, 19). The relative contributions of LECs and Myo/Nog cells to the posterior capsule and ECM deposition after cataract surgery can be revisited with cell type specific markers and probes for mRNA.

Myo/Nog cells are considered the source of myofibroblasts in the lens which we define as single nucleated, contractile cells that synthesize skeletal muscle proteins in addition to α-SMA. Myo/Nog cells are inherently myogenic, express multiple striated muscle proteins as well as α-SMA, and contract to deform the capsule (8–10, 18). Treatment that specifically kills Myo/Nog cells in human lens explants and the rabbit lens during cataract surgery nearly prevents the emergence of myofibroblasts (8–10). In the rabbit lens, targeting Myo/Nog cells also resulted in a dramatic decrease in deformations of the posterior capsule and a reduction in the severity of PCO, ACO and Soemmering’s rings to below clinically significant levels (10). Furthermore, there was a paucity of cells on the post-surgical posterior capsule in the absence of Myo/Nog cells and myofibroblasts. The argument in favor of Myo/Nog cells as the source of contractile myofibroblasts is also supported by α-SMA’s inability to mediate contraction of LECs and their lack of striated muscle protein expression (8, 73). However, α-SMA is likely to be important for fortifying stress fibers for cell adhesion and migration (71, 84–86).

Myo/Nog cells are present in all normal and diseased tissues examined thus far (7). They rapidly multiply and migrate to wounds and dying cells in the embryo and adult (8, 10, 20–22, 24, 25, 87, 88). Myofibroblasts derived from the liver, kidney and lung also express MyoD (64, 67, 69), and Noggin is produced in myofibroblasts of the colon (89). Given Myo/Nog cells’ inherent capacity to synthesize muscle proteins, it is likely that they are a source of myofibroblasts throughout the body.

Depletion of Myo/Nog cells with BAI1ab:3DNA:Dox was effective in preventing the accumulation of myofibroblasts and mitigating PCO without off target effects in the avascular lens (10). This approach may also target myofibroblasts that continue to express BAI1 and Noggin. Eliminating Myo/Nog cells and their differentiated progeny in other organs via systemic drug injection may reduce myofibroblast-mediated pathology. However, adverse effects may result from a loss of Myo/Nog cells’ titration of BMP signaling and phagocytosis (13, 19, 24). Depending on the approach, temporary blockage of Myo/Nog cell differentiation may expand the progenitor population poised to generate myofibroblasts, as demonstrated in this study. Determination of the bioavailability of targeting drugs in acute and chronic diseases, the sensitivity of quiescent Myo/Nog cells in normal tissues to cytotoxic molecules and the extent to which they repopulate organs after depletion are necessary areas for exploration when considering a therapeutic approach that targets this population to prevent abnormal traction in the lens and other tissues.

## Data Availability

The datasets presented in this study can be found in online repositories. The names of the repository/repositories and accession number(s) can be found in the article/supplementary material.
